# Supplementary material for: Fungi Tryptophan Synthases: What Is the Role of the Linker Connecting the α and β Structural Domains in Hemileia vastatrix TRPS? A Molecular Dynamics Investigation
Source: Molecules. 2024 Feb 6;29(4):756. doi: 10.3390/molecules29040756 (PMC10893352; doi:10.3390/molecules29040756)

Supplement Figure S1: Multiple sequence alignment between TRPS from Melampsora larici, Puccinia striiformis, Puccinia sorghi, Puccinia graminis, Puccinia triticina and the Hemileia vastatrix protein contig_208623_14257. The alignment was built by Muscle.

* (asterisk) indicates positions which have a single, fully conserved residue.

: (colon) indicates conservation between groups of strongly similar properties - scoring > 0.5 in the Gonnet PAM 250 matrix.

. (period) indicates conservation between groups of weakly similar properties - scoring =< 0.5 in the Gonnet PAM 250 matrix.

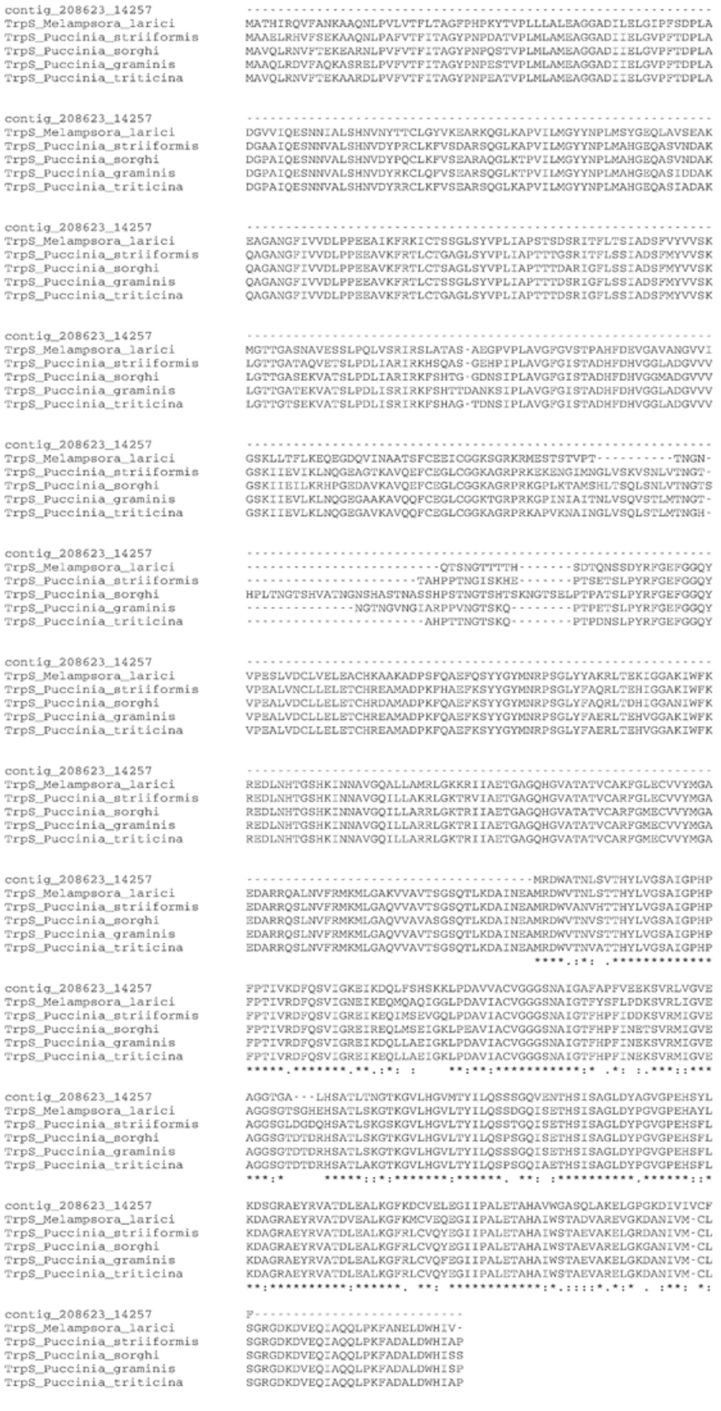

Supplement Figure S2: Multiple sequence alignment between TRPS from *Melampsora larici*, *Puccinia striiformis*, *Puccinia sorghi*, *Puccinia graminis*, *Puccinia triticina* and the newly annotated *Hemileia vastatrix* Tryptophan synthase. The alignment was built by Muscle.

* indicates positions which have a single, fully conserved residue.

: indicates conservation between groups of strongly similar properties - scoring > 0.5 in the Gonnet PAM 250 matrix.

. indicates conservation between groups of weakly similar properties - scoring =< 0.5 in the Gonnet PAM 250 matrix.

```
TrpS_Hemileia_vastatrix        MSADIKRVFATNSQLNIPVFISFVTAGFPHPDLTVPILLALQAAGSHIIELGIPFTDPLA
TrpS_Melampsora_larici         MATHIRQVFANKAAQNLPVLVTFLTAGFPHPKYTVPLLLALEAGGADILELGIPFSDPLA
TrpS_Puccinia_striiformis      MAAELRHVFSEKAAQNLPAFVTFITAGYPNPDATVPLMLAMEAGGADIIELGVPFTDPLA
TrpS_Puccinia_sorghi           MAVQLRNVFTEKEARNLPVFVTFITAGYPNPQSTVPLMLAMEAGGADIIELGVPFTDPLA
TrpS_Puccinia_graminis         MAAQLRDVFAQKASRELPVFVTFITAGYPNPESTVPLMLAMEAGGADIIELGVPFTDPLA
TrpS_Puccinia_triticina        MAVQLRNVFTEKAARDLPVFVTFITAGYPNPEATVPLMLAMEAGGADIIELGVPFTDPLA
                               *:.  :.  **:  :     ::*.:::*:***:*:*.  ***::**::*.*:  *:***:**:****

TrpS_Hemileia_vastatrix        DGPAIQSANNVALKHNVSYKDTLNLLSLARQRGLSVPVVLMGYYNPFLAHGEAQAVVDAK
TrpS_Melampsora_larici         DGVVIQESNNIALSHNVNYTTCLGYVKEARKQGLKAPVILMGYYNPLMSYGEQLAVSEAK
TrpS_Puccinia_striiformis      DGAAIQESNNVALSHNVDYPRCLKFVSDARSQGLKAPVILMGYYNPLMAHGEQASVNDAK
TrpS_Puccinia_sorghi           DGPAIQESNNVALSHNVDYPQCLKFVSEARAQGLKTPVILMGYYNPLMAHGEQASVNDAK
TrpS_Puccinia_graminis         DGPAIQESNNVALSHNVDYRKCLQFVSEARSQGLKTPVILMGYYNPLMAHGEQASIDDAK
TrpS_Puccinia_triticina        DGPAIQESNNVALSHNVDYRRCLKFVSEARSQGLKAPVILMGYYNPLMAHGEQASIADAK
                               ** .**.:**:**.***.*    *   :.  **  .**..**:*******::::**  :: :**

TrpS_Hemileia_vastatrix        KARANGFIIVDLPPEEATTFRQYCTDHQMSYIPLIAPSTPDHRIEFIKSIADSFVYVVSK
TrpS_Melampsora_larici         EAGANGFIVVDLPPEEAIKFRKICTSSGLSYVPLIAPSTSDSRITFLTSIADSFVYVVSK
TrpS_Puccinia_striiformis      QAGANGFIVVDLPPEEAVKFRTLCTGAGLSYVPLIAPTTTGSRITFLSSIADSFMYVVSK
TrpS_Puccinia_sorghi           QAGANGFIVVDLPPEEAVKFRTLCTSAGLSYVPLIAPTTTDARIGFLSSIADSFMYVVSK
TrpS_Puccinia_graminis         QAGANGFIVVDLPPEEAVKFRTLCTSSGLSYVPLIAPTTTDSRIGFLSSIADSFMYVVSK
TrpS_Puccinia_triticina        QAGANGFIVVDLPPEEAVKFRTLCTGAGLSYVPLIAPTTTDSRIGFLSSIADSFMYVVSK
                               :* *****:********  .**   **.   :**:*****:*..  ** *:.******:*****

TrpS_Hemileia_vastatrix        MGVTGASALLEEYLPNLLQKIK-----SSGSKSPHLAVGFGISTPSHFDHVASLAEGVVV
TrpS_Melampsora_larici         MGTTGASNAVESSLPQLVSRIRSLAT-ASAEGPVPLAVGFGVSTPAHFDEVGAVANGVVI
TrpS_Puccinia_striiformis      LGTTGATAQVETSLPDLIARIRKHSQ-ASGEHPIPLAVGFGISTADHFDHVGGLADGVVV
TrpS_Puccinia_sorghi           LGTTGASEKVATSLPDLIARIRKFSH-TGGDNSIPLAVGFGISTADHFDHVGGMADGVVV
TrpS_Puccinia_graminis         LGTTGATEKVATSLPDLISRIRKFSHTTDANKSIPLAVGFGISTADHFDHVGGLADGVVV
TrpS_Puccinia_triticina        LGTTGTSEKVATSLPDLISRIRKFSH-AGTDNSIPLAVGFGISTADHFDHVGGLADGVVV
                               :*.**::  :    **:*: .*.         :. .  .   ******:**. *** *..:*:***:

TrpS_Hemileia_vastatrix        GSKLSEFFNDAP---KFDQLIQFCKTLTSNN--KPRTSPLSDL-----------------
TrpS_Melampsora_larici         GSKLLTFLKEQEGDQVINAATSFCEEICGGKSGRKRMESTSTVPT----------TNGN-
TrpS_Puccinia_striiformis      GSKIIEVIKLNQGEAGTKAVQEFCEGLCGGKAGRPRKEKENGIMNGLVSKVSNLVTNGT-
TrpS_Puccinia_sorghi           GSKIIEILKRHPGEDAVKAVQEFCEGLCGGKAGRPRKGPLKTAMSHLTSQLSNLVTNGTS
TrpS_Puccinia_graminis         GSKIIEVLKLNQGEGAAKAVQQFCEGLCGGKTGRPRKGPINIAITNLVSQVSTLMTNGT-
TrpS_Puccinia_triticina        GSKIIEVLKLNQGEGAVKAVQQFCEGLCGGKAGRPRKAPVKNAINGLVSQLSTLMTNGH-
                               ***:   .::            .    .**: :  ..:   .  *     .

TrpS_Hemileia_vastatrix        ------------------------PKLDSLNET-------MPSQTSSSSLSKISTGRFG
TrpS_Melampsora_larici         ------------------------QTSNGTTTT-------HSDTQNSSDY------RFG
TrpS_Puccinia_striiformis      ---------------------TAHPPTNGISKH-------EPTSETSLPY------RFG
TrpS_Puccinia_sorghi           HPLTNGTSHVATNGNSHASTNASSHPSTNGTSHTSKNGTSELPTPATSLPY------RFG
TrpS_Puccinia_graminis         -------------NGTNGVNGIARPPVNGTSK--------QPTPETSLPY------RFG
TrpS_Puccinia_triticina        ----------------------AHPTTNGTSK--------QPTPDNSLPY------RFG
                                                             :. .            .   .*          ***

TrpS_Hemileia_vastatrix        SFGGQYVPEIIWQCLVELEAAHYNAMHDPVFLDEFKSYYGYMNRPSQLYHATRLTQAWGG
TrpS_Melampsora_larici         EFGGQYVPESLVDCLVELEACHKAAKADPSFQAEFQSYYGYMNRPSGLYYAKRLTEKIGG
TrpS_Puccinia_striiformis      EFGGQYVPEALVNCLLELETCHREAMADPKFHAEFKSYYGYMNRPSGLYFAQRLTEHIGG
TrpS_Puccinia_sorghi           EFGGQYVPEALVDCLLELETCHRDAMADPKFQAEFKSYYGYMNRPSGLYFAQRLTDHIGG
TrpS_Puccinia_graminis         EFGGQYVPEALVDCLLELETCHREAMADPKFQAEFKSYYGYMNRPSGLYFAERLTEHVGG
TrpS_Puccinia_triticina        EFGGQYVPEALVDCLLELETCHREAMADPKFQAEFKSYYGYMNRPSGLYFAERLTEHVGG
                               .********  :  :**:***:.*   *  ** *   **:********** **.* ***:   **

TrpS_Hemileia_vastatrix        ANIWLKREDLNHTGSHKINNALGQILLAKRLGKSRIIAETGAGQHGVATATVCAKFGMEC
TrpS_Melampsora_larici         AKIWFKREDLNHTGSHKINNAVGQALLAMRLGKKRIIAETGAGQHGVATATVCAKFGLEC
TrpS_Puccinia_striiformis      AKIWFKREDLNHTGSHKINNAVGQILLAKRLGKTRVIAETGAGQHGVATATVCARFGLEC
TrpS_Puccinia_sorghi           ANIWFKREDLNHTGSHKINNAVGQILLARRLGKTRIIAETGAGQHGVATATVCARFGMEC
TrpS_Puccinia_graminis         AKIWFKREDLNHTGSHKINNAVGQILLARRLGKTRIIAETGAGQHGVATATVCARFGMEC
TrpS_Puccinia_triticina        AKIWFKREDLNHTGSHKINNAVGQILLARRLGKTRIIAETGAGQHGVATATVCARFGMEC
                               *:**:*****************:** *** ****.*:*******************.**:**

TrpS_Hemileia_vastatrix        VVYMGEEDARRQALNVFRMRMLGAKVVCVASGSRTLKDAISFVDEAMRDWATNLSVTHYL
TrpS_Melampsora_larici         VVYMGAEDARRQALNVFRMKMLGAKVVAVTSGSQTLKDAI---NEAMRDWVTNLSTTHYL
TrpS_Puccinia_striiformis      VVYMGAEDARRQSLNVFRMKMLGAQVVAVTSGSQTLKDAI---NEAMRDWVANVHTTHYL
TrpS_Puccinia_sorghi           VVYMGAEDARRQSLNVFRMKMLGAQVVAVASGSQTLKDAI---NEAMRDWVTNVSTTHYL
TrpS_Puccinia_graminis         VVYMGAEDARRQSLNVFRMKMLGAQVVAVTSGSQTLKDAI---NEAMRDWVTNVSTTHYL
TrpS_Puccinia_triticina        VVYMGAEDARRQSLNVFRMKMLGAQVVAVTSGSQTLKDAI---NEAMRDWVTNVATTHYL
                               ***** ******:*******.****:**.*:***.******   :******.:*: .****

TrpS_Hemileia_vastatrix        VGSAIGPHPFPTIVKDFQSVIGKEIKDQLFSHSKKLPDAVVACVGGGSNAIGAFAPFVEE
TrpS_Melampsora_larici         VGSAIGPHPFPTIVRDFQSVIGNEIKEQMQAQIGGLPDAVIACVGGGSNAIGTFYSFLPD
TrpS_Puccinia_striiformis      VGSAIGPHPFPTIVRDFQSVIGREIKEQIMSEVGQLPDAVIACVGGGSNAIGTFHPFIDD
TrpS_Puccinia_sorghi           VGSAIGPHPFPTIVRDFQSVIGREIREQLMSEIGKLPEAVIACVGGGSNAIGTFHPFINE
TrpS_Puccinia_graminis         VGSAIGPHPFPTIVRDFQSVIGREIKDQLLAEIGKLPDAVIACVGGGSNAIGTFHPFINE
TrpS_Puccinia_triticina        VGSAIGPHPFPTIVRDFQSVIGREIKEQLLAEIGKLPDAVIACVGGGSNAIGTFHPFINE
                               **************.*******.**.:*: :    **:**:***********:* .*: :

TrpS_Hemileia_vastatrix        KSVRLVGVEAGGTGA---LHSATLTNGTKGVLHGVMTYILQSSSGQVENTHSISAGLDYA
TrpS_Melampsora_larici         KSVRLIGVEAGGSGTSGHEHSATLSKGTKGVLHGVLTYILQSSDGQISETHSISAGLDYP
TrpS_Puccinia_striiformis      KSVRMIGVEAGGSGLDGDQHSATLSKGSKGVLHGVLTYILQSSTGQISETHSISAGLDYP
TrpS_Puccinia_sorghi           TSVRMIGVEAGGSGTDTDRHSATLSKGTKGVLHGVLTYILQSPSGQISETHSISAGLDYP
TrpS_Puccinia_graminis         KSVRMIGVEAGGSGTDTDRHSATLSKGTKGVLHGVLTYILQSSSGQISETHSISAGLDYP
TrpS_Puccinia_triticina        KSVRMIGVEAGGSGTDTDRHSATLAKGTKGVLHGVLTYILQSPSGQIAETHSISAGLDYP
                               .***::******:*      *****::*:*******:******. **: :**********.

TrpS_Hemileia_vastatrix        GVGPEHSYLKDSGRAEYRVATDLEALKGFKDCVELEGIIPALETAHAVWGASQLAKELGP
TrpS_Melampsora_larici         GVGPEHAYLKDAGRAEYRVATDVEALKGFKMCVEQEGIIPALETAHAIWSTADVAREVGK
TrpS_Puccinia_striiformis      GVGPEHSFLKDAGRAEYRVATDLEALKGFRLCVQYEGIIPALETAHAIWSTAEVAKELGR
TrpS_Puccinia_sorghi           GVGPEHSFLKDAGRAEYRVATDLEALKGFRLCVQYEGIIPALETAHAIWSTAEVARELGK
TrpS_Puccinia_graminis         GVGPEHSFLKDAGRAEYRVATDLEALKGFRLCVQFEGIIPALETAHAIWSTAEVAKELGK
TrpS_Puccinia_triticina        GVGPEHSFLKDAGRAEYRVATDLEALKGFRLCVQYEGIIPALETAHAIWSTAEVARELGK
                               ******::***:**********:******. **: ************:*.::::*.*:*

TrpS_Hemileia_vastatrix        GKDIVISFSGRGDKDVEEVSRRLPEFAKELNWHIAPEKISHGNSAS
TrpS_Melampsora_larici         DANIVMCLSGRGDKDVEQIAQQLPKFANELDWHIV----------
TrpS_Puccinia_striiformis      DANIVMCLSGRGDKDVEQIAQQLPKFADALDWHIAP---------
TrpS_Puccinia_sorghi           GANIVMCLSGRGDKDVEQIAQQLPKFADALDWHISS---------
TrpS_Puccinia_graminis         DANIVMCLSGRGDKDVEQIAQQLPKFADALDWHISP---------
TrpS_Puccinia_triticina        DANIVMCLSGRGDKDVEQIAQQLPKFADALDWHIAP---------
                               . :**:.:*********:::..**:**. *:***
```

Supplementary Figure S3: Structural superposition of 3D models for TRPS. In blue the Alphafold result for *H. Vastatrix* Tryptophan synthase. In green the Modeller 3D0 proposed structure for *H. Vastatrix* Tryptophan synthase. RMSD between pruned atoms was 1.018 Angstroms.

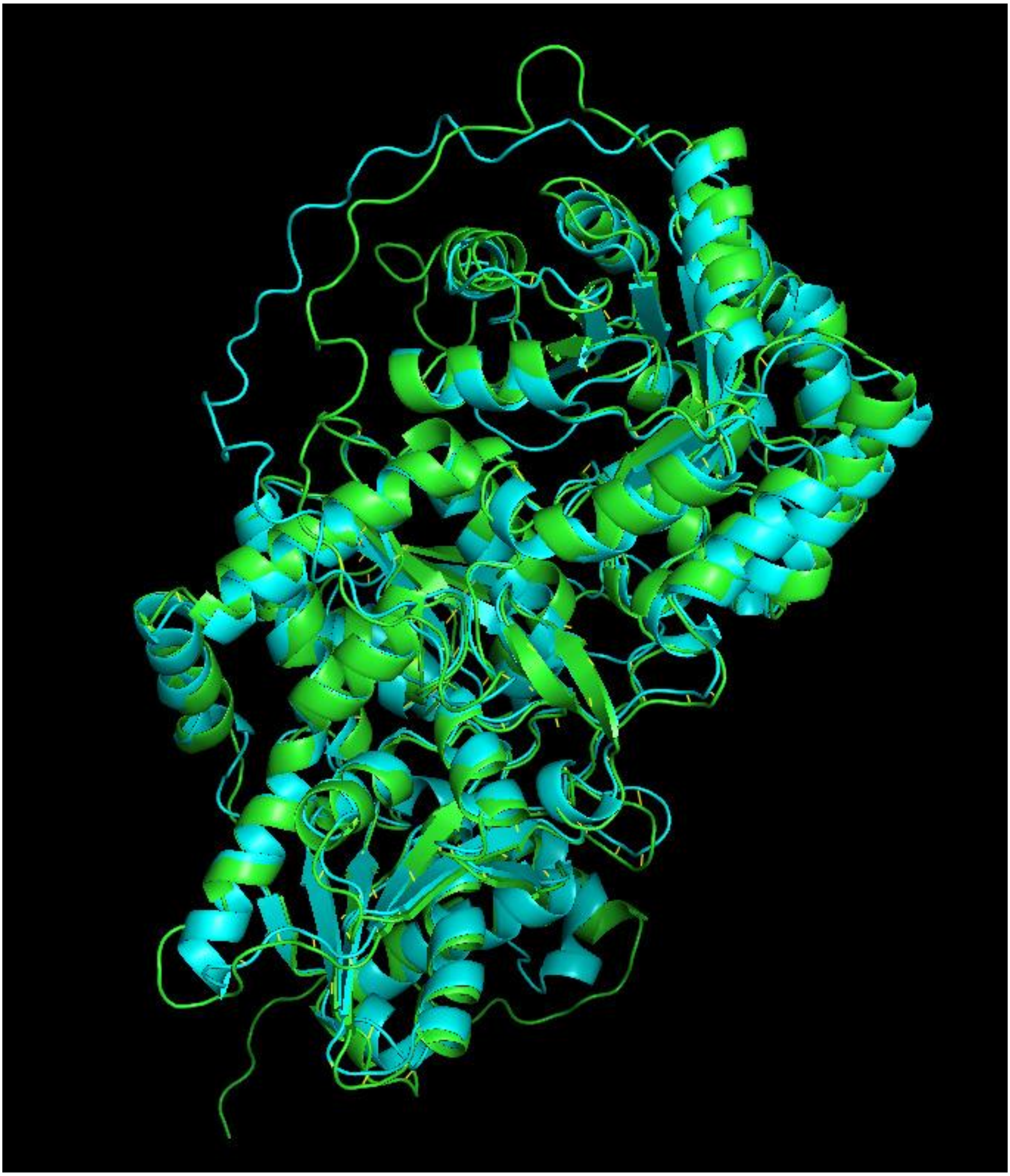

Supplement: Supplementary file 1 [file molecules-29-00756-s001.zip › molecules-2821221-supplementary.pdf]
